# Apparent diffusion coefficient correlates with different histopathological features in several intrahepatic tumors

**DOI:** 10.1007/s00330-023-09788-6

**Published:** 2023-06-22

**Authors:** Alexey Surov, Kai Ina Eger, Johann Potratz, Sebastian Gottschling, Andreas Wienke, Dörthe Jechorek

**Affiliations:** 1 Department of Radiology, Neuroradiology and Nuclear Medicine , Johannes Wesling University Hospital, Ruhr-University, Bochum, Germany; 2 Institute of Pathology, University of Magdeburg, Leipziger Str. 44, 39112 Magdeburg, Germany; 3 Department of Radiology and Nuclear Medicine, University of Magdeburg, Leipziger Str. 44, 39112 Magdeburg, Germany; 4 Institute of Medical Epidemiology, Biostatistics, and Informatics, Martin-Luther-University Halle-Wittenberg, Magdeburger Str. 8, 06097 Halle, Germany

**Keywords:** Liver, Neoplasms, Pathology, Tomography

## Abstract

**Objectives:**

To investigate associations between apparent diffusion coefficient (ADC) and cell count, Ki 67, tumor-stroma ratio (TSR), and tumoral lymphocytes in different hepatic malignancies.

**Methods:**

We identified 149 cases with performed liver biopsies: hepatocellular cancer (HCC, *n* = 53), intrahepatic cholangiocarcinoma (iCC, *n* = 29), metastases of colorectal cancer (CRC, *n* = 24), metastases of breast cancer (BC, *n* = 28), and metastases of pancreatic cancer (PC, *n* = 15). MRI was performed on a 1.5-T scanner with an axial echo-planar sequence. MRI was done before biopsy. Biopsy images of target lesions were selected. The cylindrical region of interest was placed on the ADC map of target lesions in accordance with the needle position on the biopsy images. Mean ADC values were estimated. TSR, cell counts, proliferation index Ki 67, and number of tumor-infiltrating lymphocytes were estimated. Spearman’s rank correlation coefficients and intraclass correlation coefficients were calculated.

**Results:**

Inter-reader agreement was excellent regarding the ADC measurements. In HCC, ADC correlated with cell count (*r* =  − 0.68, *p* < 0.001) and with TSR (*r* = 0.31, *p* = 0.024). In iCC, ADC correlated with TSR (*r* = 0.60, *p* < 0.001) and with cell count (*r* =  − 0.54, *p* = 0.002). In CRC metastases, ADC correlated with cell count (*r* =  − 0.54 *p* = 0.006) and with Ki 67 (*r* =  − 0.46, *p* = 0.024). In BC liver metastases, ADC correlated with TSR (*r* = 0.55, *p* < 0.002) and with Ki 67 (*r* =  − 0.51, *p* = 0.006). In PC metastases, no significant correlations were found.

**Conclusions:**

ADC correlated with tumor cellularity in HCC, iCC, and CRC liver metastases. ADC reflects TSR in BC liver metastases, HCC, and iCC. ADC cannot reflect intratumoral lymphocytes.

**Clinical relevance statement:**

The present study shows that the apparent diffusion coefficient can be used as a surrogate imaging marker for different histopathological features in several malignant hepatic lesions.

**Key Points:**

*• ADC reflects different histopathological features in several hepatic tumors.*

*• ADC correlates with tumor cellularity in HCC, iCC, and CRC metastases.*

*• ADC strongly correlates with tumor-stroma ratio in BC metastases and iCC.*

## Introduction

Magnetic resonance imaging (MRI) plays an essential role in the diagnosis and local staging of liver tumors including hepatocellular cancer (HCC), cholangiocarcinoma, and liver metastases (LM) from non-hepatic malignancies.

Besides the great diagnostic potential, MRI can also provide information regarding tumor histopathology. So far, diffusion-weighted imaging (DWI) reflects tissue microstructure [1]. DWI is related to the free movement of water molecules (Brownian molecular movement) and is quantified by means of the apparent diffusion coefficient (ADC) [1]. In tissues, free water movement is hindered predominantly by cells. According to the literature, there are inverse correlations between ADC and cellularity in several lesions [2–4]. For instance, ADC correlates well with cell count in lung cancer and neck cancer [3, 4]. Also, ADC is associated with the proliferation index Ki 67. So far, ADC correlates strongly with Ki 67 in breast cancer, lung cancer, and ovarian carcinoma [5, 6].

Finally, ADC reflects tumor grade in different cancers. For example, ADC can discriminate high and low-grade ovarian cancers [6].

In hepatic malignancies, such as hepatocellular cancer (HCC) or intrahepatic cholangiocarcinoma (iCC), DWI can also predict tumor differentiation. It was shown that DWI can predict tumor grade and microvascular invasion in HCC and iCC [7, 8]. However, in contrast to other tumors, only a few studies investigated associations between DWI/ADC and the expression of histopathological markers like Ki 67 in liver lesions [9–11]. Moreover, the reported results are mixed. In addition, there are no reports about relationships between DWI and histopathology in liver metastases.

Besides proliferation and differentiation, DWI can also differentiate tumors with high and low stromal ratios as well as tumors with high and low epithelial-mesenchymal transition [12, 13]. So far, in eosophageal cancer, ADC correlated strongly the amount of stromal collagen (*r* =  − 0.729, *p* = 0.001) [12]. Furthermore, ADC correlated with tumor stroma ratio (TSR) (*r* = 0.47, *p* = 0.007) in a murine model of rhabdomyosarcoma [13]. In liver tumors, no previous studies analyzed this question.

Recently, some reports indicated that DWI can also reflect the number of intratumoral immune cells [14, 15]. For example, ADC correlated well with tumor-infiltrating lymphocytes in breast cancer [15].

Therefore, the aim of the present study was to investigate possible associations between ADC and expression of Ki 67, cell count, TSR, and tumoral immune cells in different hepatic malignancies.

## Materials and methods

This retrospective study was approved by the institutional review board (Medical Faculty, Otto-von-Guericke-University Magdeburg, number 43/20).

###  Data acquisition and patients

We retrospectively reviewed the radiological database for liver biopsies performed in our department in the time period from 2012 to 2021 (computerized search for key codes of the reports). Overall, 1256 cases were identified. In the next step, the imaging and histopathological findings of the identified cases were analyzed. The inclusion criteria for the study were as follows:

- Documented position of biopsy needle on CT/MR images
- Available MRI with ADC images before biopsy
- Lesion size > 5 mm
- Available pathologic specimens

The exclusion criteria were as follows:

- Cases, which did not meet inclusion criteria
- Significant artifacts of ADC images

**Fig. 1:**
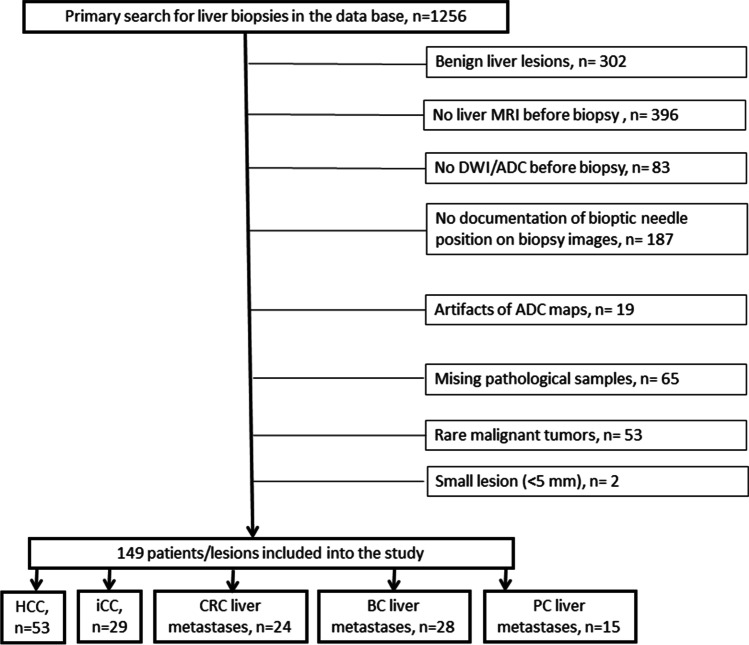
Flow chart of data acquisition. HCC, hepatocellular cancer; iCC, intrahepatic cholangiocarcinoma; BC, breast cancer; CRC, colorectal cancer, PC, pancreatic cancer

Overall, 1107 cases were excluded (Fig. 1). The remaining 149 patients met the inclusion criteria. There were 68 (45.6%) women and 81 men (54.4%) with a mean age of 65.3 ± 12.3 years and a median age of 64.5 years.

The included patients had different malignant intrahepatic tumors. In 53 patients (35.6%), hepatocellular cancer (HCC) was diagnosed, in 29 patients (19.5%), intrahepatic cholangiocarcinoma (iCC), in 24 patients (16.1%), liver metastases of colorectal cancer (CRC) were identified, in 28 patients (18.8%), liver metastases of breast cancer, and in 15 patient liver metastases of pancreatic adenocarcinoma (PC) (10.0%) were found.

### Imaging

In all patients, MRI was performed on a 1.5-T clinical scanner (Achieva, Philips Healthcare). The imaging protocol included T2-weighted single-shot and turbo-spin echo sequences with and without fat suppression (TR/TE: 1600:100). Dynamic contrast-enhanced scans were obtained after administration of Gd-EOB-DTPA (0.1 mmol/kg body weight, Primovist®, Bayer HealthCare): T1 weighted gradient echo sequences in the arterial, portal-venous and late venous phase as well as hepatobiliary imaging about 20 min after contrast application (TR/TE: 4:2). Axial diffusion-weighted echo-planar imaging sequence with respiratory triggering (TR/TE: 1959/59, FoV: 360 × 360, matrix 144 × 142, b values of 0 and 600 s/mm^2^, one repetition per b value for b0 and 4 repetitions per b value for b600, flip angle 90) was obtained. ADC maps were automatically generated by the software.

Biopsies were performed under sterile conditions by using 18-G coaxial needles. In every case, two to three cylindrical specimens were sampled.

### Imaging analysis

**Fig. 2:**
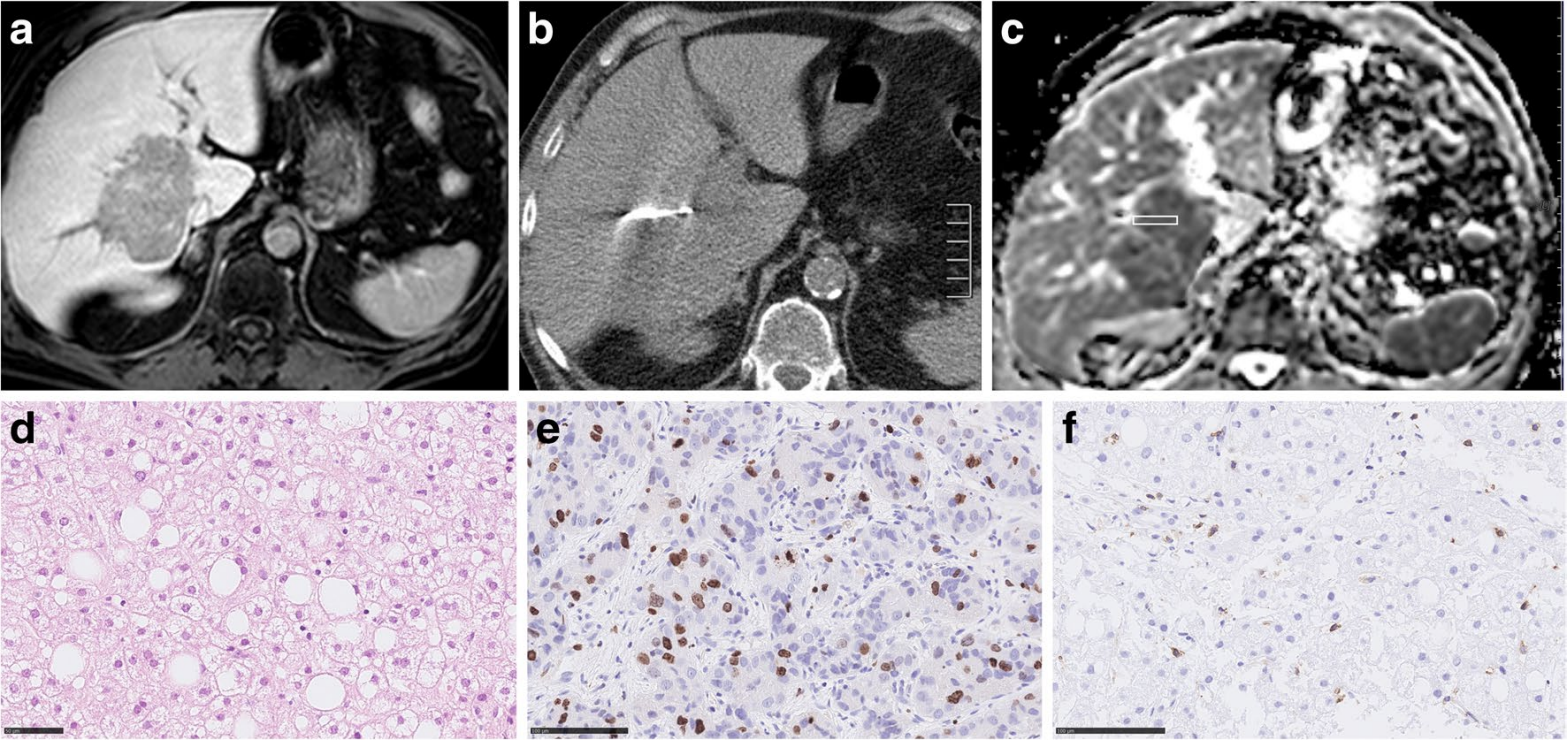
Imaging and histopathological features of a hepatocellular cancer lesion. **a** MR image of the lesion (venous phase). **b** CT-guided biopsy image with documentation of needle position within the target lesion. **c** ADC image of the biopsied lesion with the cylindrical region of interest corresponding with the needle position on CT biopsy images. The estimated mean ADC value is 0.68 × 10^–3^ mm.^2^/s. **d** H&E-stained image. Cell count is 151. The mean stromal ratio is 8%. **e** MIB-stained image. Ki 67 index is 40%. **f** CD 45 stained image. The mean number of CD 45 positive cells is 5

**Fig. 3:**
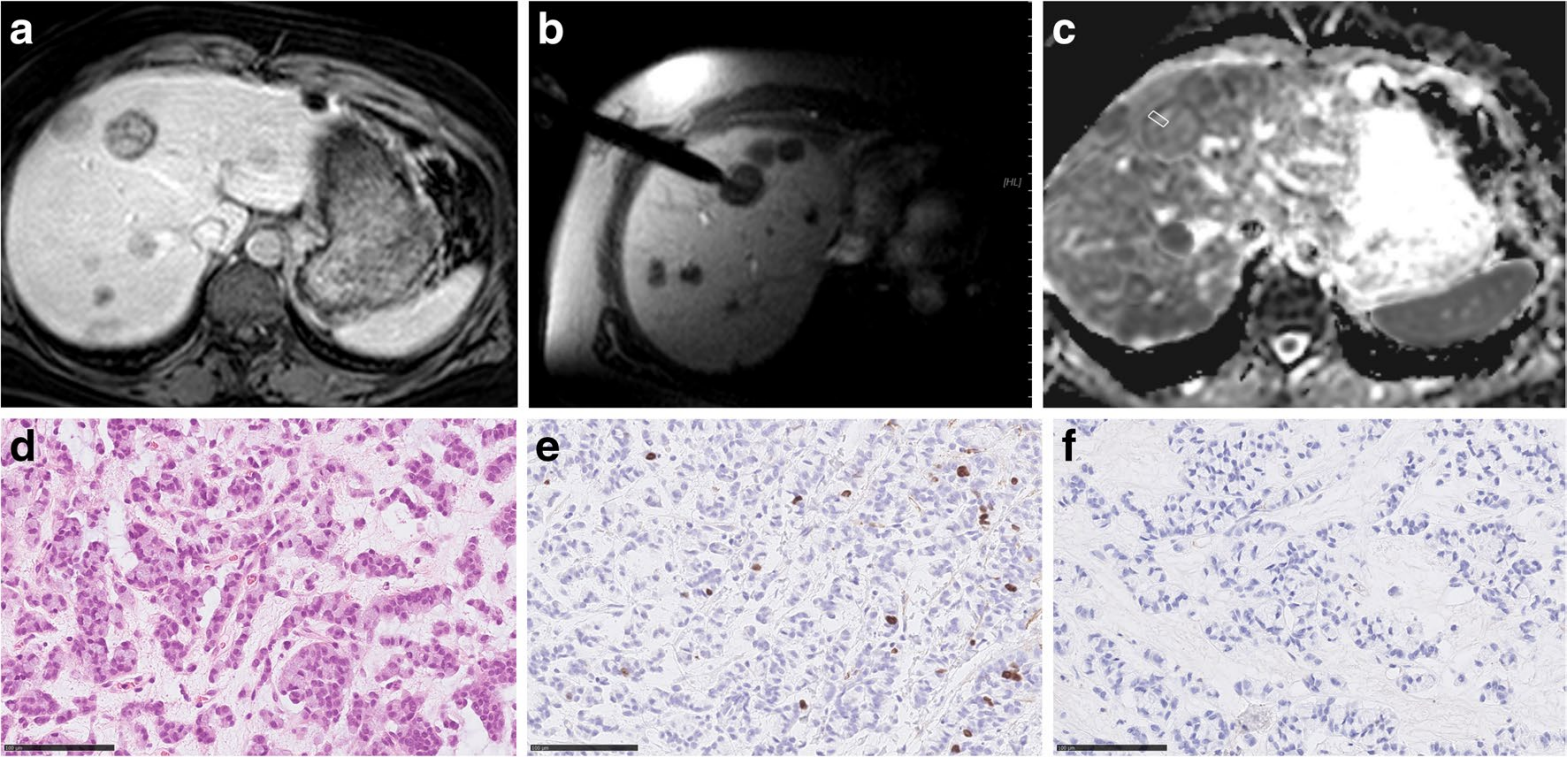
Imaging and histopathological features of breast cancer liver metastases. **a** MR image documenting lesion (venous phase). **b** MR-guided biopsy image with documentation of needle position within the target lesion. **c** ADC image of the target lesion with the cylindrical region of interest corresponding with the needle position on MR biopsy images. The estimated mean ADC value is 1.1 × 10^–3^ mm.^2^/s. **d** H&E-stained image. Cell count is 197. The mean stromal ratio is 22%. **e** MIB-stained image. Ki 67 index is 6%. **f** CD 45 stained image. The mean number of CD 45 positive cells is 1

**Fig. 4:**
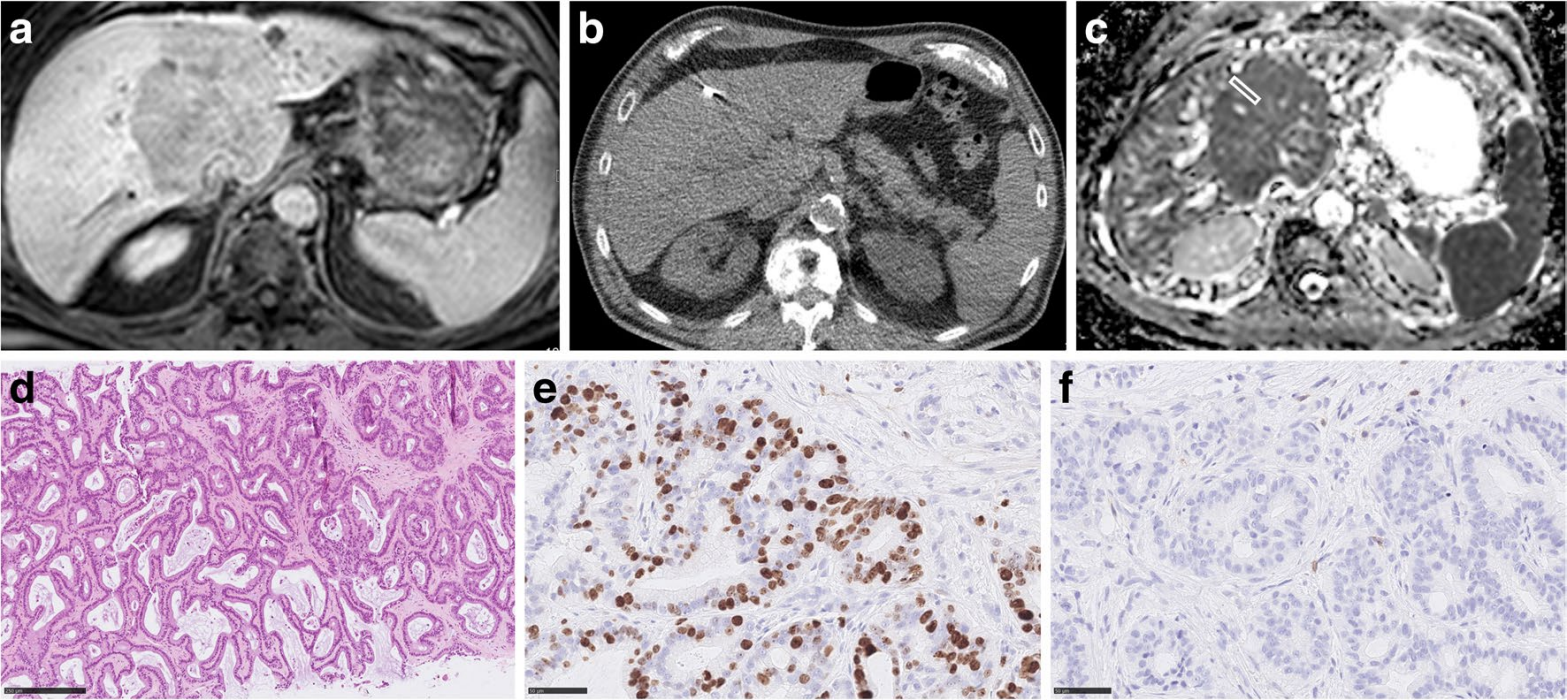
Imaging and histopathological features of intrahepatic cholangiocarcinoma. **a** Target lesion on T1w image (venous phase). **b** CT-guided biopsy image with documentation of needle position within the target lesion. **c** ADC image of the target lesion with the cylindrical region of interest corresponding with the needle position on CT biopsy images. The estimated mean ADC value is 0.84 × 10^–3^ mm.^2^/s. **d** H&E-stained image. Cell count is 272. TSR is 54%. **e** MIB-stained image. Ki 67 index is 35%. **f** CD 45 stained image. The mean number of CD 45 positive cells is 3

**Fig. 5:**
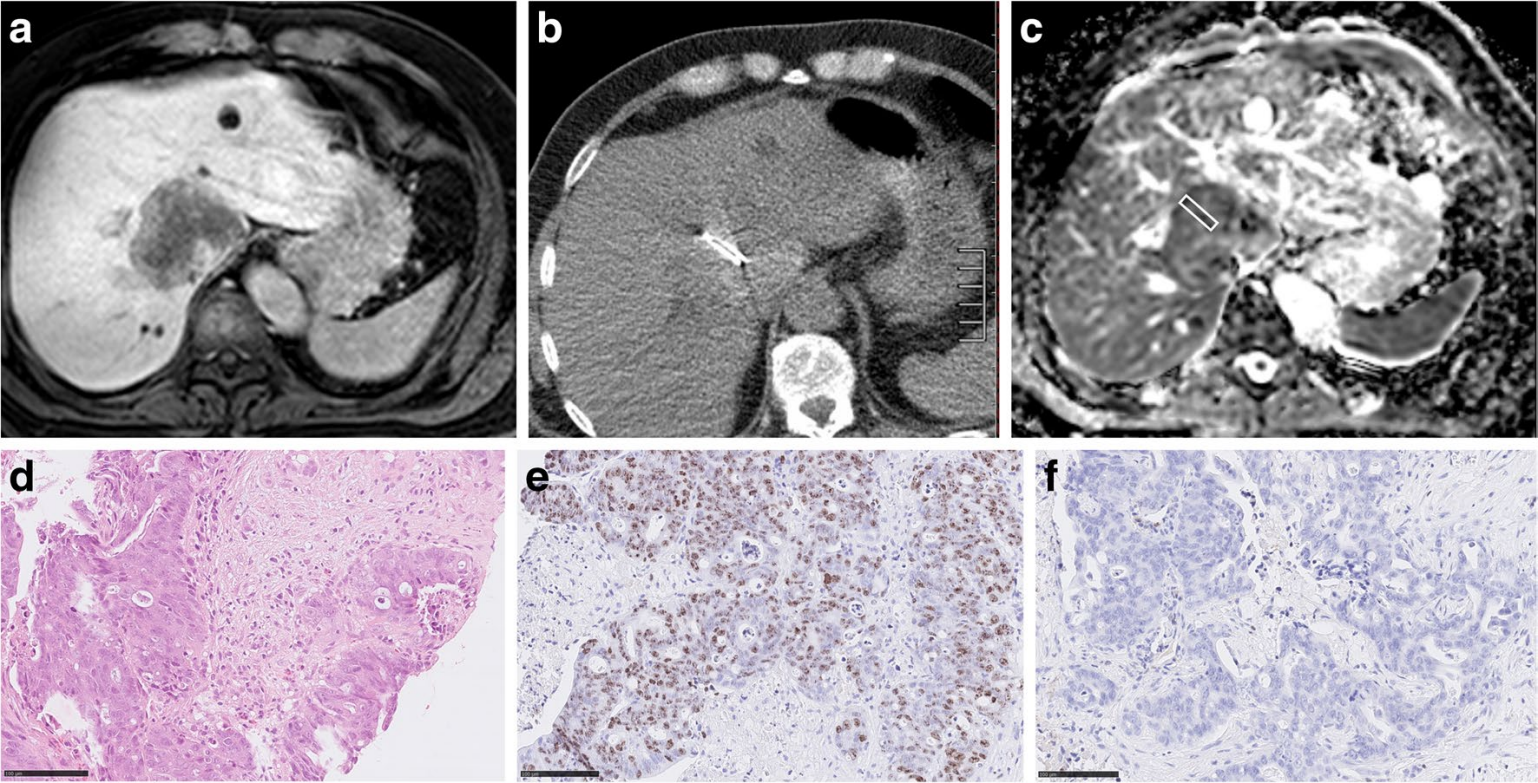
Associations between ADC and histopathological features in colorectal cancer liver metastases. **a** Target lesion on T1w image (venous phase). **b** CT-guided biopsy image with documentation of needle position within the target lesion. **c** ADC image of the target lesion with cylindrical region of interest corresponding with the needle position on CT biopsy images. The estimated mean ADC value is 0.90 × 10^–3^ mm.^2^/s. **d** H&E-stained image. Cell count is 308. TSR is 30%. **e** MIB-stained image. Ki 67 index is 90%. **f** CD 45 stained image. The mean number of CD 45 positive cells is 2

**Fig. 6:**
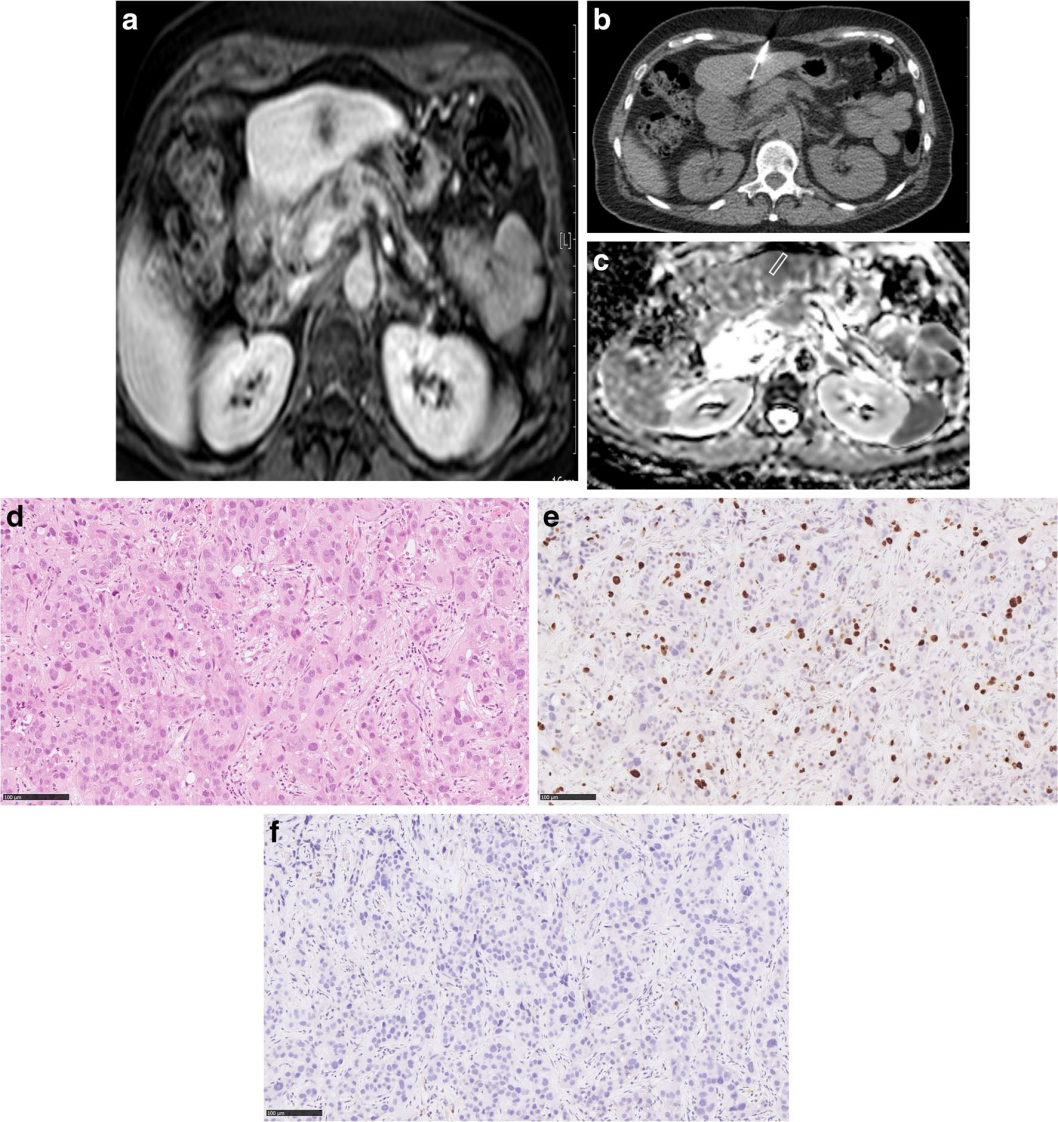
Relationships between ADC and histopathological features in pancreatic cancer liver metastases. **a** Target lesion on T1w image (venous phase). **b** Documentation of needle position within the target lesion on CT-guided biopsy image. **c** ADC image of the target lesion with the cylindrical region of interest according to the needle position on CT biopsy images. The estimated mean ADC value is 1.05 × 10^–3^ mm^2^/s. **d** H&E-stained image. Cell count is 113. TSR is 5.5%. **e** MIB-stained image. Ki 67 index is 20%. **f** CD 45 stained image. The mean number of CD 45 positive cells is 1.5

Only the MR images before the biopsy were considered for the study. Measurements were performed on a Picture Archiving and Communication System (PACS) workstation (Infinitt, Version 3.0, Infinitt Healthcare). In the first step, in every case, biopsy images with documentation of needle position were analyzed and the target lesion was determined. In the second step, a cylindrical region of interest (ROI) was placed on the ADC map of every target lesion exactly in accordance with the needle position on the biopsy images. ADC maps were co-registered with the post-contrast T1-weighted 3D-gradient echo sequence to improve visualization and correlation. Analysis of images was performed by two experienced radiologists (S.G. with 2 years of experience in hepatobiliary MRI and A.S., board-certified radiologist, with 16 years of experience in hepatobiliary MRI), independent of each other and blinded to the histopathological findings of the lesions. For all lesions mean ADC values were estimated. Figures 2, 3, 4, 5, and 6 show examples of the analyzed tumor entities in the present investigations.

### Histopathological analysis

The biopsies of the tumors were obtained before any form of treatment. Histopathology was evaluated by two experienced investigators (D.J., K.E.) in consensus without knowledge of the patients or imaging data.

Formalin-fixed, paraffin-embedded tissue serial Sects. (2 µm) were dewaxed in xylol and rehydrated by descending concentrations of ethanol. For each specimen, standard hematoxylin and eosin (HE) staining and immunohistochemistry were performed. For antigen detection, we used the automated immunohistochemistry slide staining system VENTANA BenchMark ULTRA (Roche Diagnostics GmbH), the VENTANA iVIEW DAB Detection Kit (Roche Diagnostics GmbH), and the indirect biotin-streptavidin method before counterstaining with Haemalaun solution. Antigen retrieval was performed with CC1mild, followed by incubation with specific primary antibodies recognizing CD45/leucocyte common antigen (polyclonal mouse antibody, clone 2B11 + PD7/26; DAKO/Agilent #M0701) or Ki67 (polyclonal mouse antibody, clone Mib1; DAKO/Agilent #M7240), at 36 °C for 32 min, dilution 1:500 or 1:100, respectively.

Every histopathological parameter was evaluated in five power fields (× 40; 0.23 mm^2^ per field). For each specimen, the mean values of the quantified parameter were calculated. Tumor-stroma ratio (TSR) was evaluated on the HE-stained specimen and percentages were given per tumor and stroma content separately. Cell density or density of tumor-infiltrating immune cells were estimated as a mean of overall cell counts or CD45 + leucocytes per high power field, respectively. The rate of proliferation was indicated by the percentage of Ki 67-positive cells from all tumor cells (Ki 67-index). Histopathological evaluation was done with the Nikon ECLIPSE Ni-E microscope. Pictures were digitalized with the camera Nikon DS-Ri2 and saved as uncompressed Tagged Image File Format (TIFF).

### Statistical analysis

Statistical analysis was performed using the SPSS package (IBM SPSS Statistics for Windows, version 28: IBM corporation, 2021). The collected data were evaluated by means of descriptive statistics (absolute and relative frequencies). A comparison of ADC values and histopathological parameters in groups was performed by ANOVA tests. The correlation between ADC and histopathological features was calculated by Spearman’s rank correlation coefficient. Intraclass correlation coefficients (ICC) were used for the calculation of interreader variability. *p* values were interpreted in an exploratory manner.

## Results

### Comparison of ADC values and histopathological findings in the analyzed tumors

The measured ADC values showed a good interreader variability both for the overall sample and for the subgroups, ranging from ICC = 0.815 to ICC = 0.981 (Table 1).

**Table 1:** Interobserver agreement of ADC values

	Interobserver agreement
Total sample	0.946 *p* < 0.001
HCC	0.976 *p* < 0.001
iCC	0.959 *p* < 0.001
BC liver metastases	0.981 *p* < 0.001
CRC liver metastases	0.913 *p* < 0.001
PC liver metastases	0.815 *p* = 0.001

*HCC*, hepatocellular cancer; *iCC*, intrahepatic cholangiocarcinoma; *BC*, breast cancer; *CRC*, colorectal cancer, *PC*, pancreatic cancer

In the overall sample, the mean ADC value (× 10^–3^ mm^2^/s) was 0.93 ± 0.30, median value, of 0.86, range, 0.45–2.00. ADC values in the subgroups are shown in Table 2. ADC values were lowest in PC metastases. The highest ADC values were identified in iCC.

**Table 2:** Comparison of ADC values and histopathological features in the analyzed tumors

	HCC	CC	CRC liver metastases	BC liver metastases	PC liver metastases	*p* values*
ADC, × 10^–3^ mm^2^/s	0.96 ± 0.28	1.03 ± 0.38	0.94 ± 0.33	0.86 ± 0.19	0.75 ± 0.22	0.023

*HCC*, hepatocellular cancer; *CC*, cholangiocarcinoma; *BC*, breast cancer; *CRC*, colorectal cancer; *PC*, pancreatic cancer

^*^ ANOVA

A comparison of histopathological parameters in the subgroups is shown in Table 3. TSR was lowest in HCC and highest in iCC. There was no significant difference in regard to cell count between the investigated tumors. HCC showed the highest number of CD 45 positive cells. The lowest number of CD 45 positive cells was identified in CRC liver metastases. Finally, the highest proliferation index Ki 67 was found in CRC liver metastases followed by PC liver metastases.

**Table 3:** Histopathological parameters

Histopathological features	HCC *n* = 53	CC *n* = 29	CRC liver metastases *n* = 24	BC liver metastases *n* = 28	PC liver metastases *n* = 15	*p* values*
Tumor-stroma ratio, %	17.1 ± 20.0	46.5 ± 20.8	44.9 ± 24.6	38.4 ± 24.2	27.1 ± 20.8	< 0.001
Cell count, *n*	148.8 ± 59.8	158.7 ± 50.1	180.5 ± 74.6	181.9 ± 77.2	162.8 ± 54.2	0.142
CD 45, *n*	7.42 ± 7.97	4.04 ± 10.25	1.69 ± 3.00	2.80 ± 5.76	3.14 ± 4.58	0.009
Ki 67, %	17.4 ± 16.3	26.6 ± 18.6	49.2 ± 26.6	26.5 ± 15.9	38.8 ± 16.8	< 0.001

^*^ ANOVA

### Correlation between ADC values and histopathology

In the overall sample, ADC correlated moderately with cell count and slightly with Ki 67 and TSR (Table 4).

**Table 4:** Correlations between ADC values and histopathological findings in liver tumors. Total sample

	Tumor-stroma ratio	Cell count	CD 45	Ki 67
ADC	**0.293 (< 0.001)**	** − 0.518 (< 0.001)**	− 0.043 (0.602)	** − 0.293 (< 0.001)**

Significant correlations are given in boldface

In the subgroups, different correlations were found. In HCC, ADC correlated well with cell count (*r* =  − 0.68, *p* < 0.001) and slightly with TSR (*r* = 0.31, *p* = 0.024) (Table 5). In iCC, ADC correlated with TSR (*r* = 0.60, *p* < 0.001) and with cell count (*r* =  − 0.54, *p* = 0.002) (Table 6). In CRC metastases, ADC correlated with cell count (*r* =  − 0.54 *p* = 0.006) and with Ki 67 (*r* =  − 0.46, *p* = 0.024) (Table 7). In BC liver metastases, ADC correlated with TSR (*r* = 0.55, *p* < 0.002) and with Ki 67 (*r* =  − 0.51, *p* = 0.006) (Table 8). In PC metastases, no significant correlations were found (Table 9).

**Table 5:** Correlations between ADC values and histopathological findings in liver tumors. HCC

	Tumor-stroma ratio	Cell count	CD 45	Ki 67
ADC	**0.310 (0.024)**	** − 0.683 (< 0.001)**	− 0.154 (0.271)	− 0.267 (0.053)

Significant correlations are given in boldface

**Table 6:** Correlations between ADC values and histopathological findings in liver tumors. iCC

	Tumor-stroma ratio	Cell count	CD 45	Ki 67
ADC	**0.600 (< 0.001)**	** − 0.541 (0.002)**	− 0.171 (0.374)	− 0.183 (0.342)

Significant correlations are given in boldface

**Table 7:** Correlations between ADC values and histopathological findings in liver tumors. CRC liver metastases

	Tumor-stroma ratio	Cell count	CD 45	Ki 67
ADC	0.038 (0.859)	** − 0.540 (0.006)**	0.247 (0.245)	** − 0.458 (0.024)**

Significant correlations are given in boldface

**Table 8:** Correlations between ADC values and histopathological findings in liver tumors. BC liver metastases

	Tumor-stroma ratio	Cell count	CD 45	Ki 67
ADC	**0.551 (0.002)**	− 0.369 (0.053)	0.050 (0.802)	** − 0.510 (0.006)**

Significant correlations are given in boldface

**Table 9:** Correlations between ADC values and histopathological findings in liver tumors. PC liver metastases

	Tumor-stroma ratio	Cell count	CD 45	Ki 67
ADC	0.045 (0.873)	− 0.422 (0.117)	0.401 (0.155)	− 0.095 (0.746)

Significant correlations are given in boldface

## Discussion

To the best of our knowledge, this is the first study regarding associations between ADC and different histopathological features in several liver tumors. Based on the present results, it can be postulated that MR imaging, namely DWI can characterize different hepatic lesions. Furthermore, DWI is a valid and robust instrument. As shown, all ADC values had an excellent inter-observer agreement. This finding supports previous investigations. In fact, Kakite et al found a good inter-observer agreement of ADC values in hepatocellular cancer [16]. Similar results were found in cholagiocarcinoma [17] and CRC liver metastases [18].

Some previous reports indicated that ADC can predict tumor behavior in several liver malignancies. So far, in HCC, pretreatment low ADC, namely values under 1.32 × 10^–3^ mm^2^/s, independently predicted poorer prognosis in patients with solitary large HCCs who have undergone transcatheter arterial chemoembolization (TACE) immediately combined with radiofrequency ablation [19]. Furthermore, low minimum ADC has been reported as a risk factor for HCC recurrence following liver transplantation (20). In iCC, an increase in ADC values (< 25%) during treatment has been reported as a sign of good prognosis [21]. Also in colorectal liver metastases, ADC can predict response to systemic chemotherapy [22].

These associations may be explained by possible links between histopathology and DWI findings. Hypothetically, ADC is associated with relevant histopathological features in several liver tumors. Our results confirmed this hypothesis. Furthermore, associations between ADC values and histopathological features are different in several malignant liver lesions. In HCC, ADC correlated strongly with cell count and tended to correlate with Ki 67. Therefore, in HCC, ADC can be used as a surrogate marker for tumor cellularity. Therefore, ADC can well document the effects of cytoreductive treatments like transarterial chemotherapy, and systemic chemotherapy. This finding support also previous clinical observations. For example, according to Kostek et al, advanced HCC with a favorable response to sorafenib had a significant increase in ADC values at the first radiological evaluation after treatment [24]. In fact, an increase in ADC values suggests a decrease in cellularity and, therefore, can be used in clinical practice as a marker of treatment response. Previously, only two studies analyzed relationships between ADC and the expression of Ki 67 in HCC [9, 10]. In the study of Hu et al, ADC correlated slightly with Ki 67 (*r* =  − 0.40, *p* = 0.02) [10]. Similar results were reported by Huang et al [9]. We observed only a correlation trend between ADC and the expression of Ki 67. Previously, no studies analyzed associations between ADC and TSR as well as intratumoral lymphocytes in HCC.

Furthermore, ADC reflects cell count in iCC. Therefore, ADC can also be used for monitoring cytoreductive treatment in this tumor entity. More importantly, the present study identified that in iCC, ADC can predict TSR. According to the literature, in iCC, TSR plays an important prognostic role. So far, high TSR is inversely correlated with vascular invasion (62.5% vs 95.7%, *p* = 0.006) [25]. Furthermore, rich tumor stroma is associated with poor overall survival in patients with iCC [26]. Therefore, ADC can also be used as a prognostic marker in iCC. Our study confirms previous results of Yamada et al [27]. According to the authors, tumoral ADC correlates with TSR, and, more importantly, tumoral ADC values < 1.5 × 10–3 mm^2^/s can be used as an independent biomarker of worse overall survival [27].

In CRC liver metastases, ADC correlates with tumor cellularity and proliferation potential but not with TSR. This is in agreement with the published data [26, 27]. This finding indicates that in CRC liver metastases ADC can quantitatively reflect treatment response to chemotherapy.

In BC liver metastases, ADC correlated with Ki 67. This finding is not unusual. Previously, some reports observed significant correlations between ADC and the expression of Ki 67 in primary breast cancer [28]. Furthermore, ADC can also reflect TSR. This finding is very important. According to the literature, stroma plays an important prognostic role in breast cancer [29]. Also, Ki 67 is an essential prognostic factor in BC [28]. Therefore, ADC can also predict the prognosis of BC liver metastases.

Interestingly, in PC liver metastases, ADC did not predict relevant histopathological features. Previously, only two studies analyzed associations between ADC and histopathology in pancreatic adenocarcinomas [32, 33]. Xie et al did not find statistically significant correlations between ADC and grades of differentiation, fibrosis grade, Ki 67 expression, and expression microvessel density [32]. However, in the study of Peerboccus et al, ADC correlated significantly with tumor fibrosis and nuclear density [33]. Our finding suggests that ADC cannot be used as an imaging biomarker in liver metastases of pancreatic adenocarcinoma. Furthermore, ADC also cannot be used for monitoring cytoreductive treatment.

We could not find significant correlations between the number of intratumoral immune cells and ADC values in the investigated intrahepatic malignancies. This fact suggests that ADC cannot be used for the prediction of tumoral immune reactions. To the best of my knowledge, this is the first study analyzing relationships between DWI and intratumoral immune cells in different liver tumors.

There are several limitations of the present study. First, it is retrospective. Second, small patient samples were investigated. Clearly, further prospective studies should be performed to prove our preliminary results.

Importantly, in contrast to previous investigations, in the present study, for the first time, a measure of ADC was performed exactly on the biopsied tumor area. In other studies, a whole lesion measure of ADC values was performed, whereas histopathological analysis was made based on bioptic specimens. This results in spatial incongruences between histopathological and radiological tumor architecture. Furthermore, for the first time, associations between ADC values and complex histopathological features including tumor cell count, proliferation index, number of intratumoral lymphocytes, and tumoral stromal compartment were performed. These facts improve the significance of the present study.

In conclusion, associations between DWI and histopathology are complex and different in several hepatic malignancies. In HCC, iCC, and CRC liver metastases, ADC can be used as a surrogate marker for cell count. In BC liver metastases and iCC, ADC correlates with TSR. In BC and CRC liver metastases, ADC correlates with tumoral proliferation activity. In PC liver metastases, ADC does not reflect the analyzed histopathological features.

## Abbreviations

ADC: Apparent diffusion coefficient
BC: Breast cancer
CRC: Colorectal cancer
DWI: Diffusion-weighted imaging
HCC: Hepatocellular cancer
iCC: Intrahepatic cholangiocarcinoma
MRI: Magnetic resonance imaging
PC: Pancreatic cancer

## Declarations

### Guarantor

The scientific guarantor of this study is Alexey Surov.

### Statistics and biometry

One author has significant statistical expertise (Andreas Wienke).

### Study subjects or cohorts overlap

No study subjects or cohorts have been previously reported.

### Methodology

- retrospective
- observational
- performed at one institution
